# Derivation and characterization of matched cell lines from primary and recurrent serous ovarian cancer

**DOI:** 10.1186/1471-2407-12-379

**Published:** 2012-08-29

**Authors:** Isabelle J Létourneau, Michael CJ Quinn, Lu-Lin Wang, Lise Portelance, Katia Y Caceres, Louis Cyr, Nathalie Delvoye, Liliane Meunier, Manon de Ladurantaye, Zhen Shen, Suzanna L Arcand, Patricia N Tonin, Diane M Provencher, Anne-Marie Mes-Masson

**Affiliations:** 1 Centre de recherche du Centre hospitalier de l’Université de Montréal (CHUM)/Institut du cancer de Montréal, Montréal, Canada; 2 The Research Institute of the McGill University Health Centre, Montréal, Canada; 3 Department of Human Genetics, McGill University, Montréal, Canada; 4 Department of Medicine, McGill University, Montréal, Canada; 5 Division de gynécologie oncologique, Département d’obstétrique et gynécologie, Université de Montréal, Montréal, Canada; 6 Département de médecine, Université de Montréal, Montréal, Canada

**Keywords:** Ovarian cancer, Cell line model, Chemotherapy, Ascites

## Abstract

**Background:**

Cell line models have proven to be effective tools to investigate a variety of ovarian cancer features. Due to the limited number of cell lines, particularly of the serous subtype, the heterogeneity of the disease, and the lack of cell lines that model disease progression, there is a need to further develop cell line resources available for research. This study describes nine cell lines derived from three ovarian cancer cases that were established at initial diagnosis and at subsequent relapse after chemotherapy.

**Methods:**

The cell lines from three women diagnosed with high-grade serous ovarian cancer (1369, 2295 and 3133) were derived from solid tumor (TOV) and ascites (OV), at specific time points at diagnosis and relapse (R). Primary treatment was a combination of paclitaxel/carboplatin (1369, 3133), or cisplatin/topotecan (2295). Second line treatment included doxorubicin, gemcitabine and topotecan. In addition to molecular characterization (p53, HER2), the cell lines were characterized based on cell growth characteristics including spheroid growth, migration potential, and anchorage independence. The *in vivo* tumorigenicity potential of the cell lines was measured. Response to paclitaxel and carboplatin was assessed using a clonogenic assay.

**Results:**

All cell lines had either a nonsense or missense TP53 mutations. The ability to form compact spheroids or aggregates was observed in six of nine cell lines. Limited ability for migration and anchorage independence was observed. The OV3133(R) cell line, formed tumors at subcutaneous sites in SCID mice. Based on IC_50_ values and dose response curves, there was clear evidence of acquired resistance to carboplatin for TOV2295(R) and OV2295(R2) cell lines.

**Conclusion:**

The study identified nine new high-grade serous ovarian cancer cell lines, derived before and after chemotherapy that provides a unique resource for investigating the evolution of this common histopathological subtype of ovarian cancer.

## Background

Epithelial ovarian cancer (EOC) is the most lethal gynecological malignancy. The most common histopathogical subtype, serous, accounts for at least 50% of EOC [1,2]. Ovarian cancer grade ranges from low (highly differentiated) to high (poorly differentiated). Stage (I-IV) is classified according to the degree of spread of the disease with stage I confined to the ovaries, and stage IV associated with distant metastases. A particular feature of EOC is the formation of ascites, a peritoneal fluid with a cellular fraction of ovarian cancer cells, lymphocytes, and mesothelial cells [3,4]. While serous EOC was initially described as derived from the ovarian surface epithelia, there is a growing debate that the cancer may originate from the fallopian epithelia [5-7]. EOC is largely asymptomatic, is most frequently diagnosed at stages III-IV where the five year survival rate is typically only 30% [6]. Treatment options for EOC involve cytoreductive surgery and a combination of cisplatin/taxol as a first line of chemotherapy [8]. For early stage disease, progression free survival is determined as the end point, whereas for recurrent cancer, symptom control and quality of life are the primary treatment goal [9]. Chemotherapy response is often determined by a combination of CA-125 levels, and imaging methods such as MRI and CT scans [10,11].

Cell line models have proven to be effective tools in ovarian cancer research and have been utilized to investigate the molecular and cellular features of ovarian cancer [5,12]. We have demonstrated that EOC cell lines derived from spontaneous growth of tumor cells in culture retain many of the growth and molecular genetic characteristics of the original tumor [13]. Using ovarian cancer cell lines we have derived, we have investigated gene expression [14-18], chromosome content [19] and gene mutations [13,19]. They have also been a resource to study growth properties such as invasion and proliferation [13,20,21]. Despite the usefulness of the available cell lines, the serous subtype is under represented and there is a need for additional ovarian cancer cell lines to address the heterogeneity of the disease. Furthermore, few cell lines have been derived from treatment naïve patients, and often the resource is derived from patients that have undergone rounds of chemotherapy. In order to fully appreciate the disease and its evolution, it would be beneficial to derive cell lines from the same patient both at presentation and during the course of the disease. To date, only one report has described such a resource, exclusively derived from high grade serous ovarian ascites, where cisplatin alone was the first line therapy [22].

This study evaluated ovarian cancer cell lines derived from the same patient at diagnosis and at relapse following exposure to chemotherapy. The cell lines were developed from solid tumors and ascites. Nine cell lines were developed from specimens obtained from three patients diagnosed with high-grade serous ovarian cancer. The cell lines were characterized biologically by growth rates, morphology, ability of forming three-dimensional spheroids, migration and invasion potential, and their *in vivo* capacity to form tumors in SCID mice. Mutational status for genes important in serous EOC such as TP53, BRCA1 and BRCA2 were investigated. Information on disease progression and treatment regimens are included. In addition, we have characterized the chemosensitivity of the cell lines to paclitaxel and carboplatin by clonogenic assays. These cell lines provide novel and comprehensive models for the study of EOC progression, ascites formation and resistance to chemotherapy.

## Methods

### Patient and sample data

Tumor and ascites samples were collected with informed consent from the Centre hospitalier de l’Université de Montréal (CHUM), Hôpital Notre-Dame, in the Department of Gynecologic Oncology. The study was approved by the Comité dé’thique de la recherché du CHUM, the institutional ethics committee. Stage was determined at time of surgery by a gynecologic oncologist. Histopathology and tumor grade were determined by pathology using criteria consistent with the International Federation of Gynecology and Obstetrics (FIGO) classification [23]).

### Cell line establishment and culture conditions

In total, nine cell lines were derived from three patients. The patients were coded with the unique identifiers 1369, 2295 and 3133. All cell lines were maintained in hypoxic condition of 5% O_2_, and 5% CO_2_ and grown in complete OSE medium, which includes OSE medium (Wisent, Montreal, QC), 10% FBS, 0.5 μg/mL amphotericin B (Wisent, Montreal, QC) and 50 μg/mL gentamicin (Gibco, Grand Island, NY). The solid ovarian tumor (TOV) derived cell lines (TOV1369, TOV2295(R), TOV3133G and TOV3133D) were established using the scrape method as previously described [19,24,25]. Briefly, tumor tissue was scraped into a 100 mm plate with complete OSE medium and maintained for 40 days with the medium replaced weekly. Cells were passaged at near confluence, and were considered immortal when passaged over 50 times. The OV cell lines (OV1369(R2), OV2295, OV2295(R2), OV3133(R), OV3133(R2)) were established from the cellular fraction of ascites collected by centrifugation [25]. The cell lines derived from ascites cells were maintained as above for the TOV derived cell lines. Each cell line has reached greater than 100 passages, with the exception of TOV3133D (84 passages). All growth characteristic assays, were conducted on cell lines at passages between 60 and 80.

### Cell Growth rates

Growth rates were assessed as previously described [13,19]. Briefly, cells were seeded on day 0 in 6-well plates (1 x 10^5^ cells per well for the 1369 and 2295 cell lines and 2 x 10^5^ cells per well for 3133 cell lines). The OSE complete media was replaced every 3 days for the duration of the experiment. Every second day from day 1 to 13, cells were trypsinized, resuspended in media and counted using the CASY analyzer system. Saturation density was defined as the mean maximum number of cells counted at the time of confluence. Each experiment was performed in duplicate, and repeated three times. Doubling times were determined using a publically available algorithm [26].

### Antibodies

Western Blot (WB) and immunohistochemistry (IHC) analyses were performed using the following antibodies: beta actin (AB 6276, Abcam, Cambridge UK); TP53 (D0-1) (sc-126, Santa Cruz Biotechnology, CA, USA); HER2/ErbB2/Neu (C-18) (sc-284, Santa Cruz Biotechnology); Keratin 7 Ab-2 (MS-1352-P, NeoMarker, Medicorp, Qc, Canada); Keratin 8 Ab-4 (MS-997-P, NeoMarker, Medicorp); Keratin 18 (DC-10) (sc-6259, Santa Cruz Biotechnology); Keratin 19 Ab-1 (MS198-P, Lab Vision Corp., CA, USA), and Keratin 20 (SPM140) (ab15205, Abcam).

### Immunohistochemistry

Tissue sections were fixed in formalin and embedded in paraffin blocks. Sections (4 μm thick) were cut and slides were stained using the immunoperoxidase method. Tissue sections were heated (37°C overnight or 60°C for 30 minutes), deparaffinized in toluene and rehydrated in ascending concentrations of ethanol. Slides were then heated in boiling citrate buffer (0.01 M citric acid, pH 6.0) to unmask antigens. A 0.3% H_2_O_2_ treatment was used to eliminate endogenous peroxidase activity. The sections were blocked with a protein blocking serum-free reagent (DakoCytomation, ON, Canada) and incubated with the antibody used for western blotting for 60 min at room temperature. Tissues were incubated with a secondary biotinylated antibody (DakoCytomation) for 20 min followed by incubation with a streptavidin-peroxidase complex (DakoCytomation) for 20 min at room temperature. Staining was visualized using diaminobenzidine containing a peroxidase substrate (DakoCytomation). Hematoxylin was used as the counterstain and all sections were observed by light microscopy and pictures were taken at 40x magnification. Substitution of the primary antibody with phosphate buffered saline served as a negative control.

### Mutation analysis

DNA was extracted from cell lines as described previously [25]. TP53 mutations were initially detected by single-strand conformation polymorphism (SSCP) analysis of exons 5 to 9 of TP53 as described [19]. Band-shifts were confirmed by Sanger sequencing analysis (McGill University and Génome Québec Innovation Centre, Montréal, Québec, Canada). Samples negative by SSCP analysis were subsequently sequenced by Sanger sequencing for the coding exons 2–11. Mutation hotspots in BRAF (exon 11, exon 15) and KRAS (exon 2) were analyzed by either SSCP or sequencing as described [19]. Sequence chromatograms were compared with NCBI reference sequences reported in GenBank: NM_000546.4 (TP53), NM_004985.3 (KRAS) and NM_004333.4 (BRAF). In addition, TP53 variants were evaluated based on information in the International Agency for Research on Cancer (IARC) TP53 Database [27]. As the ovarian cancer specimens were derived from French Canadian women, a population known to exhibit founder effects and harbor recurrent BRCA1 and BRCA2 mutations [28], peripheral blood lymphocytes from each patient was investigated for the most common mutations in BRCA1 (4446C > T and 2953delGTAinsC) and BRCA2 (8765delAG, 6085 G > T and 3398delAAAAG) as previously described [28].

### Spheroid assay

A spheroid assay was conducted to determine the ability of cell lines to generate three-dimensional structures in the form of aggregates, as previously described [20,21]. Briefly, 4 × 10^3^ cells were suspended in 16 μl of complete OSE medium and placed on the cover of non-coated plastic tissue culture plates that were subsequently inverted. Phosphate buffered saline (1 x PBS) was added to the bottom plate to prevent dehydration of droplets. Spheroid formation ability was assessed in complete OSE medium after four days of incubation at 37°C, 5% O_2_, 5% CO_2_, with spheroid formation of the cell lines being classified concordant with previous research [19,21].

### Anchorage independent growth in soft agar

Cell lines were assayed for their ability to grow in anchorage independent conditions by culturing 2 x 10^4^ cells in a semi-solid media containing noble agar. Cells were included in the top layer formed of 0.33% w/v agar in complete OSE medium that was applied over a base layer (0.66% w/v agar in complete OSE medium) [25]. Cells were cultured in soft agar for three weeks, and then colonies that formed were photographed and counted. Two independent experiments were performed in triplicate.

### Wound-healing assay

Migration potential was evaluated using the scratch assay method as previously described [3,19,29]. Cells were grown to confluence in 6-well culture plate dishes. Using a 200 μl pipette tip, a wound was produced in the monolayer at different positions. The adherent monolayer was washed with 1x PBS to remove non-adherent cells and complete OSE media was then added. The same scratch was followed over time and photographed at different time points (0 h, 8 h, 24 h, 48 h). All experiments were conducted in triplicate and repeated at least twice.

### *In vivo* growth in SCID mice

The tumorigenic potential of cell lines was assessed based on their ability to form tumors in 50 day-old female SCID (severe combined immunodeficiency) mice, and NOD SCID (Charles River Laboratories, Saint-Constant, QC) at subcutaneous left gluteal injection sites. A volume of 200 μl was injected in each mouse and consisted of 5 × 10^6^ cells resuspended in 100 μl of cold phosphate buffered saline (D-PBS) (Gibco™, Invitrogen, Burlington, ON) and 100 μl of Matrigel (Becton-Dickinson, Franklin Lakes, NJ). The animals were housed under sterile conditions in a laminar flow environment with *ad libitum* access to food and water. Tumor formation was assessed twice a week for over 100 days. Animals were sacrificed before neoplastic masses reached limit points established by the Institutional Committee on Animal Protection (CIPA) according to the Canadian Council on Animal Care.

### Clonogenic assay

Chemotherapy sensitivity of cell lines was assessed using a clonogenic assay [30]. Briefly, cells were seeded in a 6-well dish at a number of cells/well that was determined to allow the formation of individual colonies (TOV1369, 500 cells/well; OV1369(R2), 200 cells/well; OV2295, 1 × 10^3^ cells/well; OV2295(R2), 2 × 10^3^ cells/well; TOV2295(R), 2 × 10^3^ cells/well; TOV3133G, 2.5 x 10^3^ cells/well; TOV3133D, 1 × 10^3^ cells/well; OV3133(R), 1 × 10^4^ cells/well; OV3133(R2), 5 × 10^4^ cells/well). Cells were seeded and allowed to adhere for 16 hours in a 37°C, 5% CO_2_, 5% O_2_ incubator after which the media was removed and replaced with OSE complete media containing paclitaxel (0–300 nM), or carboplatin (0–300 μM) (McKesson Canada, Saint-Laurent, Qc, Ca). Cells were incubated with the drug for 24 hours. The drug was then removed, cells were washed with 1 x PBS and OSE complete media was added. Media was changed weekly until colonies were visible. Colonies were then fixed with cold methanol and colored with Giemsa (Sigma–Aldrich Inc., St. Louis, MO). Colonies were manually counted and reported as percent of control. IC_50_ values were determined using Graph Pad Prism 3 software (GraphPad Software Inc., San Diego, CA). Each individual experiment was performed in triplicate and repeated three times.

## Results

### Clinical information, cell line development and tumor tissue phenotype

**Figure 1:**
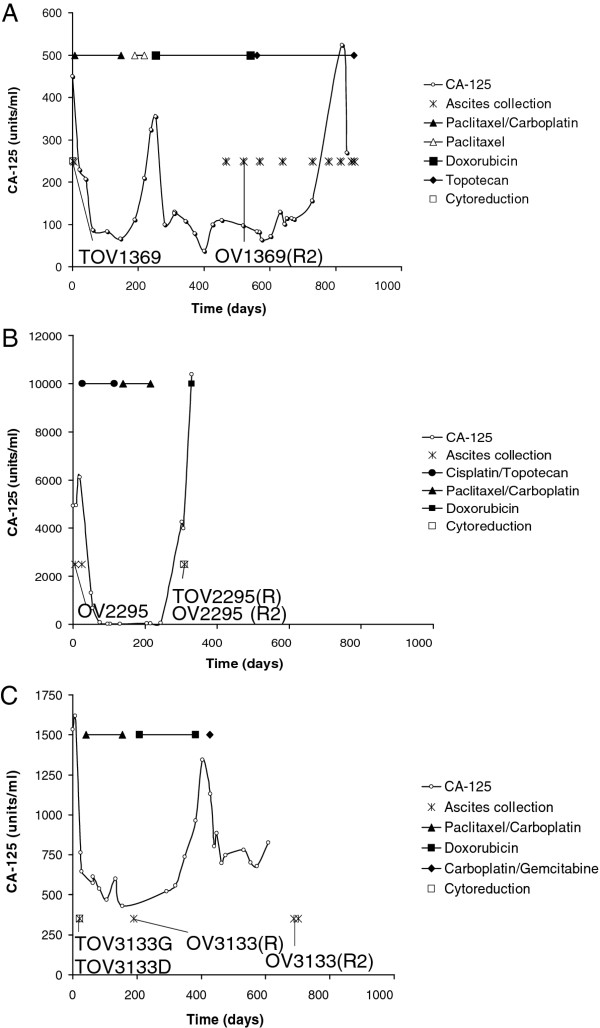
**Variation of CA-125 level over the course of the treatment of the three patients A) 1369, B) 2295 and C) 3133.** The type and length of chemotherapy treatment and time at which each cell lines were derived is also indicated. Note that the Y-axis is different for each patient.

Patient 1369 was previously diagnosed with breast cancer 18 months prior to her ovarian cancer diagnosis. Following surgical resection for breast cancer, she received docetaxel treatment, which was completed 14 months prior to her ovarian cancer diagnosis. She also entered a clinical trial, NSABP B-30 BRAS1 (doxorubicin, docetaxel with or without cyclophosphamide). She also received radiotherapy 12 months prior to her ovarian cancer diagnosis. After ovarian cancer diagnosis, she received a treatment of paclitaxel and carboplatin for 5 months. Based on the serum CA-125 levels (Figure 1), patient 1369 was initially responsive to the treatment, but due to carboplatin toxicity she was subsequently treated with paclitaxel alone. This regimen was discontinued after two cycles as CA-125 levels continued to increase. Patient 1369 had a relapse, based on X-ray computed tomography (CT), 7 months after cancer diagnosis. She then received 11 cycles of doxorubicin (month 8 to month 17). CA-125 levels decreased for the first few months following the initiation of this regimen but began to rise 6 months into the treatment (Figure 1). Topotecan was the final treatment administered from 18 to 28 months.

Patient 2295 was diagnosed with ovarian cancer following imaging, ascites puncture and partial ommentectomy. She was then included in a clinical trial, OV-16 BRAS1 (cisplatin/topotecan) for the first four months following diagnosis during which period her CA-125 levels decreased significantly, from 6000 units/ml to lower than 50 units/ml during days 96 through 200 (Figure 1B). From month four to seven, she received carboplatin and paclitaxel as part of the clinical trial OV-16 BRAS2. She first responded to the chemotherapy with a clear reduction of the tumor masses. However three months after the termination of chemotherapy, ascites volume increased and CA-125 levels increased dramatically from less than 100 units/ml at day 243 to greater than 10000 units/ml at day 330 (Figure 1B). Ten months following diagnosis, the patient underwent ovarian cytoreduction. Due to a relapse, eleven months after diagnosis, she received low doses of doxorubicin, to which she did not respond.

Patient 3133 received a treatment of paclitaxel and carboplatin one to three months after surgery and confirmation of the ovarian cancer diagnosis. CA-125 levels showed a very modest decrease during treatment (Figure 1). Imaging following the end of the chemotherapy showed many masses in the abdomen and in lymph nodes. A second surgery was performed almost six months after the first surgery, which showed an infiltration of the tumor in many areas of the abdomen. It has been concluded that the patient was resistant to her first treatment of chemotherapy. Six months after her first diagnosis, she was then put on doxorubicin for a total of five months. Again, no amelioration of the CA-125 levels was noted, and imaging detected disease evolution. The patient received carboplatin and gemcitabine 13 months after her diagnosis, for a total of 6 months. After an initial decrease in CA-125 levels from 1131 units/ml to 680 units/ml, CA-125 levels remained relatively stable (Figure 1), however imaging showed an increase in tumor mass indicating a relapse. Twenty-one months after her diagnosis, she received etoposide orally for eight days.

The cell lines were developed from samples from patients 1369, 2295 and 3133. All patients had high grade and late stage (IIIC) cystadenocarcinomas, of the serous papillary histopathology (Table 1). Patients had sub-optimal surgical debulking, and all individuals died from disease progression. The cell lines were derived from samples collected at diagnosis and at the time of relapse, from either solid tissue (TOV) or ascites (OV). In total there were four pre-chemotherapy cell lines derived from primary disease (TOV1369, OV2295, TOV3133G and TOV3133D) and five post-chemotherapy cell lines derived from recurrent (R) disease (OV1369(R2), OV2295(R2), TOV2295(R), OV3133(R) and OV3133(R2)) (Figure 1). Note that we consider TOV1369 to be a pre-chemotherapy for ovarian cancer treatment, although the patient did receive chemotherapy treatment for breast cancer 18 months prior to ovarian cancer diagnosis.

**Table 1:** Clinical data of patients 1369, 2295 and 3133 from whom the cell lines were derived

	**Patients**				
Clinical parameters	1369	2295	3133	112 ^1^	1946 ^2^
Age at diagnosis	58	59	52	42	75
Tumor type	adenocarcinoma	adenocarcinoma	adenocarcinoma	adenocarcinoma	adenocarcinoma
Histopathology sub-type	serous papillary	serous	serous papillary	endometrioid	serous papillary
Tumor grade	G3	G3	G3	G3	G3
Disease stage	IIIC	IIIC	IIIC	IIIC	IIIC
Ascites at surgery	yes	yes	yes	yes	yes
Surgical debulking	sub-optimal	sub-optimal	sub-optimal	sub-optimal	sub-optimal
Progression	yes	yes	yes	yes	yes
Death	yes	yes	yes	yes	yes
Cause of death	disease progression	disease progression	disease progression	disease progression	disease progression
Follow up (months)	28	11	22	1	0.3
Treatment	paclitaxel/carboplatin	cisplatin/topotecan	paclitaxel/carboplatin		
					
	doxorubicin	paclitaxel/carboplatin	doxorubicin		
	topotecan	doxorubicin	carboplatin/gemcitabine		
Previous history of cancer	breast cancer				
Previous chemotherapy	doxorubicine cyclophosphamide docetaxel				

^1^[13].

^2^[19] Details pertaining to patients 112 and 1946 are also included, from previous published work.

**Figure 2:**
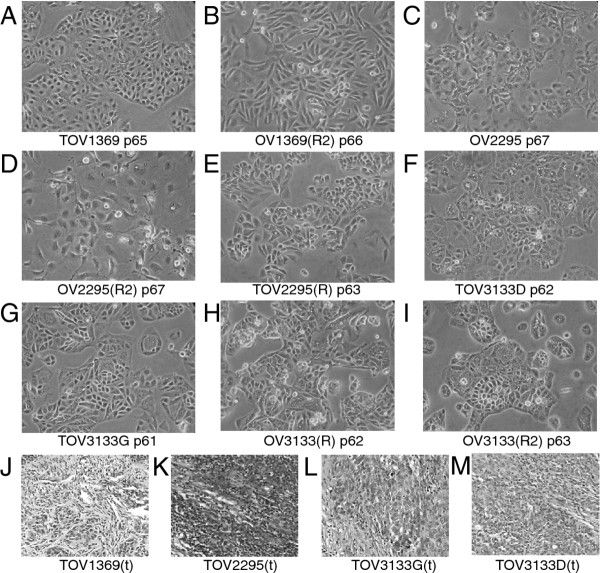
**Morphology of cell lines developed from patients 1369, 2295 and 3133 (A-I), including both primary and recurrent cell lines.** Passages were between 61 and 67 for each cell line. By the later passages, cell lines had developed into predominantly small epithelial type cells, often aggregated. There was a notable absence of fibroblast cells. Hematoxylin and eosin staining is shown in J-M for solid tumor from patients 1369, 2295 and 3133. Images are taken at x40 magnification. Note that the (t) designation denotes that the primary tumor tissue was investigated.

After 60 passages, the cell lines appeared homogeneous and no fibroblast like cells could be detected (Figure 2, A-I). Although cell shape varied for each cell line, the morphology was consistent among the lines derived from the same patient samples.

Figure 2 J to M shows hematoxylin and eosin staining of sections from the solid tumor tissue corresponding to cell line TOV1369, TOV2295(R) and TOV3133G and TOV3133D.

### Expression of keratin markers, TP53 and HER2 in tumor tissue and cell lines by Western blot and immunohistochemistry

**Figure 3:**
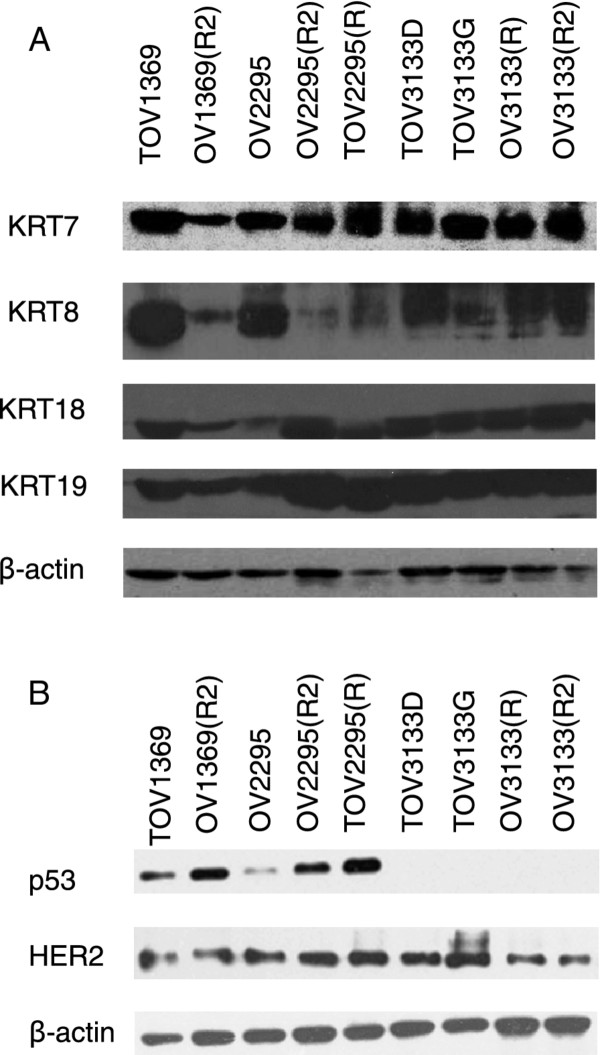
**Detection of various keratins (KRT) by Western blot (A) to confirm the epithelial origin of cell lines. B) p53 and HER2 expression by Western blot.** Note the absence of detectable expression of p53 for the 3133 cell lines. Beta actin was used as a loading control.

**Figure 4:**
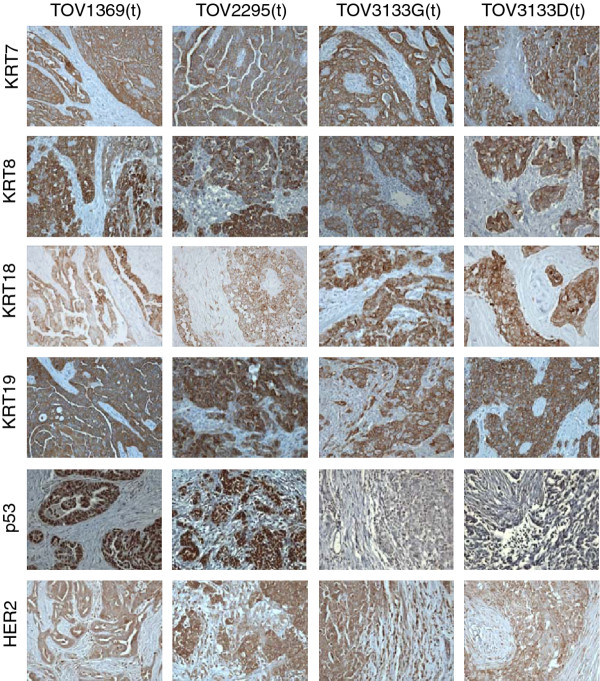
**Immunohistochemical analysis of paraffin embedded solid tumors (TOV1369, TOV2295(R), TOV3133D and TOV3133G) with keratin markers, p53 and HER20.** Nuclei are counterstained with hematoxylin, and images are at x400 magnification. Positive staining was seen for all solid tumors for each keratin tested, and HER2. Note the lower expression detected for p53 in TOV3133D and TOV3133G. Note that the (t) designation denotes that the primary tumor tissue was investigated.

In order to investigate the epithelial origin of the tumors and corresponding cell lines, keratin expression was investigated by both Western blot analysis and immmunohistochemistry. All of the keratins investigated by Western blot (keratin 7, 8, 18 and 19) were present in the protein extract of each of the nine cell lines (Figure 3A). Expression of keratins 7, 8, 18 and 19 was also observed by immunohistochemistry using sections of the solid tumor (Figure 4). Keratin 20 expression was also investigated in ovarian solid tumors (1369, 2295 and 3133) and from two colon cancer tissues, the latter being used as a positive control for keratin 20. No staining was observed in ovarian tissue, but positive staining was evident in the colon tissue ( Additional file 1). Expression of the tumor suppressor p53 was found to be present in 1369 and 2295 derived cell lines, but could not be detected in the 3133 cell lines TOV3133D, TOV3133G, OV3133(R) and OV3133(R2) (Figure 3B). Western blot results for p53 were also confirmed by immunohistochemistry, with p53 showing low expression in the TOV3133 D and TOV3133G tissues but much higher expression in TOV1369 and TOV2295(R) (Figure 4). Strong HER2 expression was detected in protein extracts of all nine cell lines (Figure 3B), and was also observed in the solid tissues by immunohistochemistry (Figure 4).

### Mutation status of TP53, BRCA1, BRCA2, KRAS and BRAF

Each of the cell lines harbored a TP53 mutation as verified by sequence analysis (Table 2). The mutation identified varied with each patient sample but were identical in the cell lines derived from the same patient samples. All variants are considered deleterious based on information from the IARC TP53 Database [27]. Although the mutations are predicted to affect the DNA binding domain, the cell lines with missense TP53 mutations tested positive for p53, whereas the cell lines with the nonsense mutation exhibited no evidence of p53 by Western blot analysis (Figure 3). The cell lines did not harbor the most commonly reported KRAS or BRAF mutations nor the most common BRCA1 and BRCA2 mutations identified in the French Canadian breast and ovarian cancer families [28]. 

**Table 2:** **Cell growth characteristics, IC**_**50**_**values and mutation status of ovarian cancer cell lines derived either at initial diagnosis (TOV1369, OV2295, TOV3133G, TOV3133D), or relapse after chemotherapy (OV1369(R2), OV2295(R2), TOV2295(R), OV3133(R), OV3133(R2))**

		**TOV1369**	**OV1369 (R2)**	**OV2295**	**OV2295 (R2)**	**TOV2295 (R)**	**TOV3133G**	**TOV3133D**	**OV3133 (R)**	**OV3133 (R2)**	**TOV112D**	**TOV1946**
Cell growth characteristic	Doubling time (avg ± SD)	2.87 ± 0.03	2.45 ± 0.12	2.90 ± 0.10	2.73 ± 0.12	2.49 ± 0.49	2.70 ± 0.15	2.63 ± 0.11	2.94 ± 0.07	3.23 ± 0.12	1.49	2.2 ± 0.26
	Saturation densisty ^1^ (x10^6^ cells) (avg ± SD)	2.37 ± 0.19	3.52 ± 0.12	1.52 ± 0.25	1.63 ± 0.12	1.46 ± 0.07	3.11 ± 0.05	3.14 ± 0.12	2.25 ± 0.25	1.34 ± 0.05	10.64	5.66 ± 0.96
	Number of passages to date	>100	>100	>100	120	>100	>100	84	>100	>100	>100	>100
Spheroid formation		no	aggregate	semi- compact	semi- compact	aggregate	semi- compact	semi- compact	no	aggregate	compact	aggregate
Migration times for wound healing (h)		>48	>48	>48	>48	>48	>48	>48	>48	>48	N/D	24
Soft agarose (colony count) ^1^ Efficiency (avg ± SD)		>0.1	8.9 ± 5.7	>.0.1	0.2 ± 0.1	0.1 ± 0.1	>0.1	5.0 ± 2.7	>0.1	6.8 ± 3.2	4.7 ± 1.4	4.7 ± 1.9
Subcutaneous injection	Number of mice with tumors	0/6	0/5	0/6	0/6	0/6	0/6	0/6	4/6	0/6	3/3	3/3
	Mean time of tumor appearance (days) ^2^	N/A	N/A	N/A	N/A	N/A	N/A	N/A	55 ± 11	N/A	>10	17 ± 4
IC_50_^3^	Carboplatin (μM) (avg ± SD)	5.64 ± 1.29	8.91 ± 5.00	0.05 ± 0.01	0.84 ± 0.25	0.93 ± 0.06	0.75 ± 0.63	1.75 ± 0.88	1.34 ± 0.37	2.65	13.96	4.04 ± 4.19
	Paclitaxel (nM) (avg ± SD)	22.8 ± 10.7	9.1 ± 6.9	5.4 ± 4.7	2.0 ± 1.5	1.9 ± 0.3	3.5 ± 0.9	2.8 ± 0.3	5.5 ± 3.2	1.6	1.9	3.5 ± 1.8
Mutation	TP53	c.730 G > T (Gly244Cys)	c.730 G > T (Gly244Cys)	c.584 T > C (Ile195Thr)	c.584 T > C (Ile195Thr)	c.584 T > C (Ile195Thr)	c.574C > T (Gln192TE)	c.574C > T (Gln192TE)	c.574C > T (Gln192TE)	c.574C > T (Gln192TE)	c.524 G > A ^4^ (Arg175His)	c.817C > T ^4^ (Arg273Cys)

^1^Calculated from two independent experiments (in triplicate);

^2^Time to tumor appearance is based on the average of the 4 mice for OV3133(R) that developed tumors, with the tumor mass being greater than 200 mm^3^;

^3^Calculated from three independent experiment except for TOV112D and OV3133(R2), which represent triplicate of a single experiment;

^4^Mutation for TP53 previously reported in Ouellet et al., 2008 [19].

### Cell growth rate and oncogenic assays

**Figure 5:**
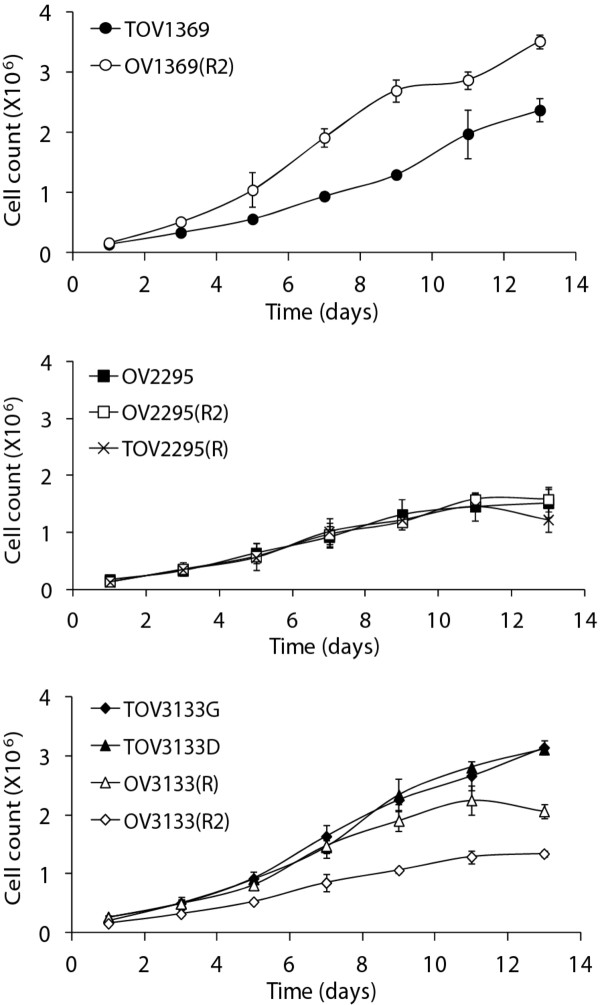
**Proliferation of cell lines over 14 days. 1X10**^**5**^** (1369 and 2295 cell lines) or 2X10**^**5**^** (3133 cell lines) cells were seeded in 6-well plates and trypsinized and counted every 48 hours, for 13 days.** Experiments were performed in duplicate, and repeated three times. Cell lines derived pre-chemotherapy are represented by an open symbol, cell lines derived post-chemotherapy have a closed symbol. Data shown is the mean ± SD.

The growth rates of the cell lines was determined (Table 2, Figure 5) and compared with TOV112D and TOV1946, cell lines previously developed by our laboratory derived from endometrioid and serous EOC respectively [19]. The proliferation of the cell lines is depicted in Figure 5. There was no difference in the doubling time for the three cell lines derived from 2295 (Table 2). OV1369(R2) had a greater proliferation rate than TOV1369. OV3133(R2) had a slower growth rate than the other 3133 cell lines (Figure 5). Similarly, the growth rates, as measured by doubling time, of all nine cell lines was slower than the pre-chemotherapy highly aggressive cell line TOV112D [19]. Doubling times ranged from 2.5 to 3.2 days, compared to 1.5 for TOV112D. There was no consistent difference in doubling between cell lines derived pre versus post chemotherapy among the three patients. Saturation densities were typically lower than what we could observed for TOV112D, being between 25% to 50% of the value obtained for TOV112D (Table 2), and more in line with the previously densities described for other serous ovarian cancer cell lines, such as TOV1946 and TOV2223 [19]. 

**Figure 6:**
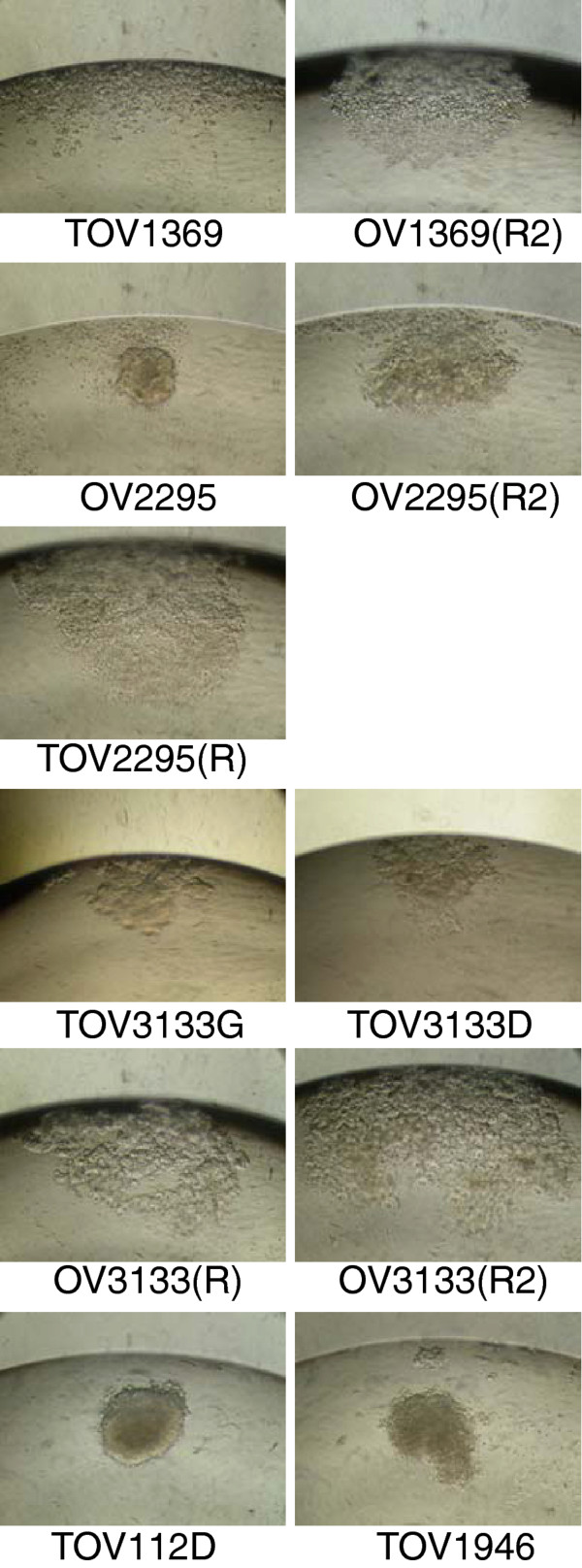
**Spheroid formation of cell lines after 4 days in OSE media using the inverted droplet technique.** Spheroid formation capability ranged from no ability (TOV1369 and OV3133(R)) to a compact spheroid (TOV112D). Photos are representative of observation from two independent experiments.

The cell line’s ability to form spheroids was measured using the hanging droplet method as previously described [21]. Although there was some variation among the replicates, none of cell lines consistently formed compact spheroids, as was clearly observed with TOV112D. Four cell lines, OV2295, OV2295(R2) TOV3133G, TOV3133D, could form semi-compact spheroids, while OV1369(R2), TOV2295(R), OV3133(R2), and TOV1946 formed numerous individual small aggregates and TOV1369 and OV3133(R) would not form spheroids (Table 2, Figure 6). 

The migration potential of the cell lines was measured using an established scratch migration assay. There were no notable differences in migration rates between the cell lines. All cell lines migrated slowly and did not fill the gap within 48 hours, which is slower than what was observed with TOV1946 [19] (see also Additional file 2).

Using the soft agarose assay, the anchorage dependency of the cells lines was investigated. After three weeks there were visible colonies formed with the OV1369(R2), OV2295(R2), TOV2295(R), TOV3133D and OV3133(R2) cell lines (Table 2).

### Xenograft tumor formation

The *in vivo* growth potential of the cell lines was determined by subcutaneous injection of cells into SCID mice (n = 6 mice for each group, with the exception of OV1369(R2) (5), TOV1946 (3) and TOV112D (3)). Only OV3133(R) formed tumors in SCID mice (66.7% of mice), whereas all other cell lines failed to induce any tumor formation (Table 2). The tumorigenic cell lines, TOV112D and TOV1946, both formed tumors in all mice. For cell line OV3133(R), the average length of tumor appearance was 55 days, which is considerably longer compared to a cell line derived from a more aggressive tumor such as TOV112D, which formed tumor within less than ten days. Also, the tumor volume formed with OV3133(R) was smaller than that derived with TOV112D ( Additional file 3) [19]. Specifically, the average volume was about 350 mm^3^ for OV3133(R) after 85 days whereas those with TOV112D and TOV1946 reached 3000 mm^3^ in less than 30 days. All other cell lines formed masses that remained at 100 mm^3^ or less for the length of the experiment ( Additional file 3). Note that on histological examination of the tumors derived from the OV3133(R) xenograft revealed an undifferentiated tumor of epithelial type cells, characteristic of high-grade serous tumors. The use of NOD-SCID mice did not appear to affect the ability of the cell lines to grow as xenografts ( Additional file 4).

### *In vitro* chemosensitivity

**Figure 7:**
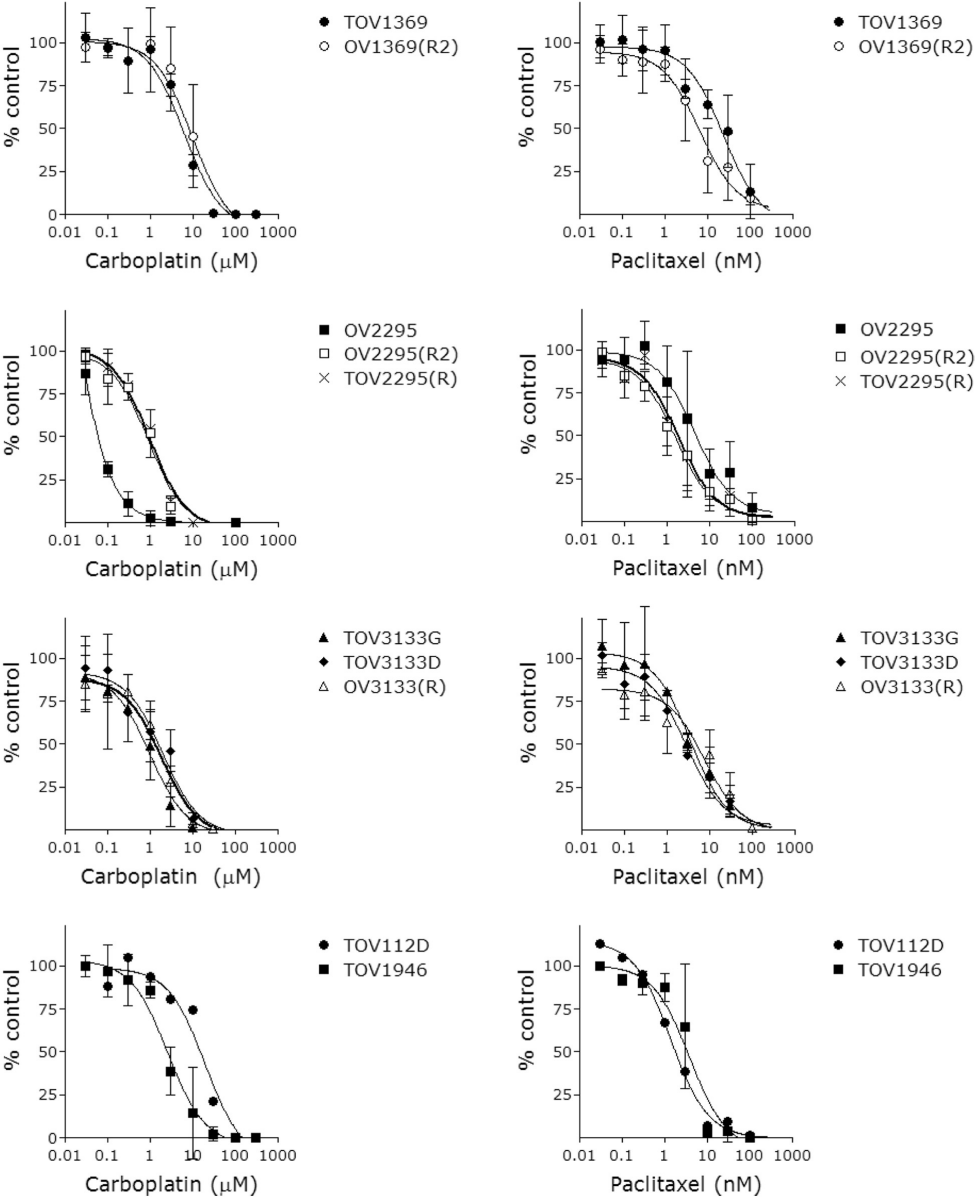
**Chemosensitivity of ovarian cancer cell lines to carboplatin and paclitaxel determined by clonogenic assay.** Log_10_ data of drug concentrations is presented versus the percentage of control. Doses ranged from 0–300 μM for carboplatin, and from 0–300 nM for paclitaxel. The graphs represent the average +/− SD of three independent experiments. For TOV112D, it is average +/− SD of a single experiment conducted in triplicate. There was a significant difference between the IC_50_ value of carboplatin for OV2295, and OV2295(R2) and TOV2295(R) (1-way ANOVA, p > 0.001).

The cell lines were investigated for their sensitivity to carboplatin and paclitaxel by determining a dose response curve (Figure 7) obtained from clonogenic assay data. The IC_50_ was calculated from these curves to allow comparison between the cell lines. Data from previously published cell lines, TOV112D and TOV1946, are included in Figure 7 for comparison.

For the 1369 cell lines (TOV1369, OV1369(R2)), IC_50_ values were much higher than other cell lines studied for carboplatin (5.6 ± 1.3 μM and 8.9 ± 5.0 μM, respectively) but more comparable to the values obtained with TOV112D and TOV1946 (Table 2, Figure 7). In the case of carboplatin, the post-chemotherapy cell line OV1369(R2) demonstrated a higher IC_50_ than the pre-chemotherapy TOV1369, but the difference was not significant (t-test p = 0.144). For paclitaxel, the IC_50_ values of TOV1369 (22.8 ± 10.7 nM) and OV1369(R2) (9.13 ± 6.88 nM) suggest a lower sensitivity to the drug when compared to the other cell lines tested (Table 2).

For 2295, the IC_50_ calculated for carboplatin for the two post-chemotherapy cell lines, OV2295(R2) and TOV2295(R) (0.84 ± 0.24 μM and 0.93 ± 0.06 μM, respectively) were more than ten fold higher than the IC_50_ of the pre-chemotherapy cell line OV2295 (0.05 ± 0.01 μM)(1-way ANOVA, p > 0.001). In the case of paclitaxel, values were similar for the three 2295 cell lines (p > 0.05) where the determined IC_50_ for OV2295, OV2295(R2) and TOV2295(R) were 5.43 ± 4.73, 1.99 ± 1.48 and 1.87 ± 0.31 nM, respectively. These results suggest that patient 2295 acquired resistance to carboplatin, but not to paclitaxel.

For the pre- and post-chemotherapy cell lines derived from patient 3133, there was no notable difference in chemosensitivity for either carboplatin or paclitaxel (Figure 7, Table 2). The IC_50_ for the 3133 cell lines ranged between 0.75 ± 0.63 μM (TOV3133G) to 2.65 μM (OV3133(R2)) for carboplatin, and from 1.59 nM (OV3133(R2)) to 5.54 ± 3.19 nM (OV3133(R)) for paclitaxel. Note that the IC_50_ value for OV3133(R2) was based on one experiment conducted in triplicate due to the low clonogenic efficiency of OV3133(R2). There was no difference in IC_50_ values between the pre- and post-chemotherapy cell lines derived from patient 3133 for either carboplatin or paclitaxel.

## Discussion

We report here on matched EOC serous cell lines derived from solid tumor or ascites samples from the same patient at time of diagnosis and following recurrence. Ovarian epithelial cells typically express keratin 7 but lack expression of keratin 20 [31-33]. This pattern was observed in the tumor tissues of all patients by both Western blot and immunohistochemistry (Figure 3; Figure 4 and Additional file 1. Taken together, we were able to confirm the epithelial origin of the cell lines presented here.

The cell lines were characterized in terms of TP53 mutation status and protein expression, BRCA1, BRCA2, KRAS and BRAF mutation status, and HER2 expression. All of the cell lines had a somatic TP53 mutation, which is consistent with the reported frequency in high-grade serous tumors, estimated at 51 to 93% in recent studies [34-37]. An identical mutation was evident in each cell line derived from the same patient, consistent with a common clonal origin for the tumor and ascites derived from each individual patient. As expected, identical TP53 mutations to those found in the cell lines were also observed in the corresponding tumor tissue (data not shown). The expression of TP53 was investigated in all of the cell lines by Western blot and in the primary solid tumors by immunohistochemistry. Interestingly, p53 was not detected by Western blot in all four 3133 cell lines (and confirmed by a low expression by immunohistochemistry). This is consistent with the truncating nonsense mutation in exon 6 in the TP53 sequence found in each of the 3133 cell lines. Expression of the epidermal growth factor receptor gene, HER2, which is implicated in malignant transformation, was also used to characterize the cell lines, as overexpression is reported on average in 20-30% of ovarian tumors and as high as 75% by a variety of techniques including ELISA, immunohistochemistry and RT-PCR [38-41]. Although there is evidence of overexpression of HER2 being associated with a lower sensitivity to platinum-based chemotherapy [41], there was no indication of differential expression in the ovarian cancer cell lines by Western blot, or in the solid tumors by immunohistochemistry that could relate to the sensitivity to carboplatin detected by the clonogenic assay.

The distinct tumor growth characteristics within the serous cell lines derived here, indicates a diversity reflective of the heterogeneous nature of this histopathological subtype. For example, saturation density, spheroid formation and colony formation in soft agarose differed between cell lines, and also within cell lines derived from the same patient. Differences in spheroid formation between cell lines derived before and after chemotherapy treatment may offer an interesting point of reference, especially as spheroid models may offer a model system more in line with the *in vivo* tumor setting [21]. For example, the cell line OV2295 formed semi-compact spheroids, compared to the aggregates in TOV2295(R), possibly reflecting differences in cell to cell adhesion. When comparing cell lines derived from a single patient over time, there is no tendency to be more aggressive in terms of the measured characteristics as the disease progresses. Nevertheless, the current model will allow researchers to address biological questions intrinsic to the cell lines such as clonal heterogeneity within tumors, as well as modification occurring for the development of ascites [42] and the relationship between biological properties of ascites and solid tumors established from the same patient [19]. In addition, the further investigation of genetic and epigenetic changes between the primary tumor and cell lines at discrete time points may provide insight into the evolutionary processes at play in cancer development [43].

Interestingly, *in vivo* tumor formation of the cell lines in SCID mice at subcutaneous sites was only observed with OV3133(R), the first ascites taken from patient 3133. The second ascites sample OV3133(R2) that was taken after doxorubicin treatment, at approximately 500 days after the OV3133(R) was sampled, did not form tumors. The tumors formed in SCID mice grew slowly as compared with the previously established TOV112D and TOV2295(R) cell lines, although this observation was consistent with previously studied high grade serous cell lines such as TOV2223 which also did not form tumors at subcutaneous sites in SCID mice [19]. Although the cell lines outlined here may not be amenable to all pre-clinical xenograft models, in the future we may be able to investigate intraperitoneal injections. Note also that the lack of tumorigenicity in mouse xenograft model may not reflect the situation in humans – this should not be used as the sole criteria for cell line utility.

The new cell lines derived in this study were developed from patient samples that were exposed to specific chemotherapeutic agents. This is in contrast to chemoresistant cell lines generated *in vitro,* which are often derived from clonal variants that survive by escalating dosages of chemotherapeutics. In contrast, the cell lines we describe here may more accurately represent the molecular evolution that occurs within the tumor microenvironment. Additionally, we have already alluded to the potential to further study the cell lines by utilizing the spheroid model, which may more accurately reflect the *in vivo* tumor environment [21]. For example, L’Esperance et al., 2008 used the spheroid model of ovarian cancer cell lines (OV90, TOV21G, TOV112D) to investigate response to chemotherapy treatment [44]. Interestingly, higher levels of both cisplatin and paclitaxel were required for a similar response in spheroids compared to a similar study using monolayers on the same cell lines [45]. This suggestion of greater drug resistance in spheroids warrants further attention in the present set of cell lines.

Of the patients from which the cell lines were established, two of the three (1369 and 2295) responded initially to first line therapy using Response Evaluation Criteria in Solid Tumors (RECIST) criteria [46,47]. In general, response rates for chemotherapy in ovarian cancer are reported at 70-80% [48]. Patient 3133 did not show a clear response to chemotherapy, with evidence of progressive disease after 5 months by RECIST criteria. However, CA-125 levels showed a marked decrease from 764 before the initial paclitaxel/carboplatin treatment to 470 units per ml two months following treatment . This decrease of nearly 40% is just outside the level of decrease which would be indicative of a responder, based on the GCIG (Gynecological Cancer InterGroup) acceptable current criteria for CA-125 response, of at least a 50% decrease in CA-125, for at least 28 days[46,47]. Although no significant differences were observed in response to paclitaxel in the cell lines derived from primary versus recurrent disease, previous studies have indicated otherwise, such as described in a recent report which determined that 35% of solid tumors and 50% of ascites samples were resistant to paclitaxel [49]. It is noteworthy that the IC_50_ levels of paclitaxel response in TOV1369 and OV1369(R2) are four-to-twenty and two-to-five times higher, respectively, than all other cell lines examined, and this is possibly due to acquired resistance to taxol as a consequence of prior treatment for breast cancer. We also note that cells from patient 1369 also displayed a lower sensitivity to carboplatin, although the clinical profile of this patient does not suggest inherent chemoresistance. Comparison of the OV2295 (derived prior to recurrence) to the OV2295(R2) and TOV2295(R) cell lines derived following recurrence were the only clear example of acquired resistance to carboplatin. Carboplatin resistance is well documented in ovarian cancer. For example, a recent study found 75% of solid tumors and 59% of ascites samples to be resistant to carboplatin [49]. This is likely due to the selective pressure of the chemotherapy regime exerted on a heterogeneous cell population, resulting in an enrichment of a resistant subset of cells by promoting the expression of a resistance pathway or selection for a population bearing a mutation responsible for a decrease in sensitivity.

Mutation status such as TP53, BRCA1 and BRCA2 [50,51] are also important factors, which may contribute to tumor progression and chemoresistance of an ovarian tumor tissue or cell line, especially in relation to their role in apoptosis. In this report, based on the investigation of common French Canadian mutations, no BRCA1 or BRCA2 mutations were identified, and therefore we cannot comment on the role of BRCA1/2 as a surrogate marker for chemotherapy response. There did not appear to be a difference in the specific type of TP53 mutation (truncating nonsense for 3133, missense for 1369 and 2295), relative to chemosensitivity status. Although there is evidence of overexpression of HER2 being associated with a lower sensitivity to platinum-based chemotherapy, our results did not show differential expression in the ovarian cancer cell lines by Western blot, or in the solid tumors by immunohistochemistry that could relate to the sensitivity to carboplatin detected by the clonogenic assay. Therefore we can suggest that BRCA and HER2 are not linked to the resistance profile presented in our cell lines and that other factors might be involved. In the case of p53, there was a difference in mutant p53 protein expression between TOV1369 and OV1369(R2), but no corresponding difference in chemotherapy response. Although the OV2295 cell line appeared to have a lower expression of the mutant p53 protein then the recurrent OV2295(R2) and TOV2295(R) cell lines, both of which exhibited acquired carboplatin resistance, any relationship between mutant p53 expression and carboplatin resistance would have to be robustly tested using a gene knock-down experiment. Furthermore, a study using paired pre- and post-chemotherapy tumor samples, determined that differences in gene expression profiles between matched samples could be due to factors not only involved in chemotherapy resistance, but also factors related to tumor progression and proliferation [44,52]. The cell lines described here may serve as a good model to begin to analyze specific candidates identified in these studies.

## Conclusion

The new ovarian cancer cell lines characterized in this report provide an important biological resource for studying the molecular genetic evolution of ovarian cancer that reflect the development of disease in the context of initial diagnosis and following disease recurrence. The cell lines provide a framework for comparative molecular genetic studies investigating the genomic landscape by gene expression, copy number variation or mutation analysis. The unique phenotypes exhibited by the cell lines suggest that they reflect the complexity of ovarian cancer disease. The paired sample cell line model from patient 2295, will allow for further discrimination of acquired resistance, affected by the chemotherapy regime. Although cell lines from patient 1369 and 3133 do not show signs of acquired resistance, both offer avenues of research into tumor evolution. Specifically, further study of the1369 cell lines would allow for the understanding of the molecular basis for the high innate resistance to both carboplatin and paclitaxel. In 3133, three distinct time points are represented offering possible insights into the mechanisms of tumor progression and evolution.

## Competing interests

The authors declare that there are no competing interests.

## Authors’ contributions

I.J.L. performed the clonogenic assays to assess chemosensitivity of the cell lines, contributed to the anchorage independent growth assay, participated in the xenograft tumor formation experiment and performed all data analysis and writing of the manuscript. M.C.J.Q. participated in all analysis, wrote the majority of the paper and was responsible for all editing of the manuscript. L.P. established the cell lines from tumor and ascites tissue. M.dL. and D.M.P. were responsible for all clinical data. L.W. performed the growth characteristic assays (proliferation assay, spheroid assay, migration assay), data analysis of the migration assay and immunohistochemistry as well as participated in the xenograft formation experiment. K.Y.C. performed the Western blot and aided with the anchorage independent growth assay. L.C. and N.D. contributed to the clonogenic assay. L.M. helped perform the anchorage independent growth assay. Z.S. and S.L.A. performed the mutation analyses under the supervision of PNT. P.N.T., D.M.P. and A-M.M-M. were involved in the study conception and design, and interpretation of the data. All authors revised the manuscript.

## Pre-publication history

The pre-publication history for this paper can be accessed here:

http://www.biomedcentral.com/1471-2407/12/379/prepub

## Supplementary Material

Additional file 1 ** Keratin 7 and keratin 20 immunohistochemistry in ovarian tumors from which the cell lines were derived.** Paraffin embedded colon tumors were used as the negative control for keratin 7, and positive control for keratin 20. Sections were counterstained with hematoxylin and are shown at x40 magnification. Click here for file

Additional file 2 ** Images from the migration assay, indicating migration of cells during a 48-hours period.** Note the closure of the wound for the TOV1946 cell line after 48 hours. Click here for file

Additional file 3 ** Tumor growth in SCID mice following injection of specific ovarian cancer cell lines.** For each cell line, six SCID mice received subcutaneous injection (5 x 10^6^ cells mixed with Matrigel) (with the exception of five mice for OV1369(R2), and three mice for TOV112D and TOV1946). Graphs represent average ± SD of tumor volume as a function of days following cell injection. Tumor masses were measured at least twice a week (width x length x thickness). Note that the axis for TOV112D and TOV1946 is not of the same scale. Cell lines derived pre-chemotherapy are represented by a closed symbol, cell lines derived post-chemotherapy have an open symbol. Click here for file

Additional file 4 ** Tumor growth in SCID-NOD mice following injection of specific ovarian cancer cell lines.** For each cell line, two NOD-SCID mice received subcutaneous injection (5 x 10^6^ cells mixed with Matrigel). Graphs represent average of tumor volume as a function of days following cell injection. Tumor masses were measured at least twice a week (width x length x thickness). Note that the axis for TOV112D and TOV1946 is not of the same scale. Cell lines derived pre-chemotherapy are represented by a closed symbol, cell lines derived post-chemotherapy have an open symbol. Click here for file
