# Supplementary figures and images for: Derivation and characterization of matched cell lines from primary and recurrent serous ovarian cancer
Source: BMC Cancer. 2012 Aug 29;12:379. doi: 10.1186/1471-2407-12-379 (PMC3532154; doi:10.1186/1471-2407-12-379)

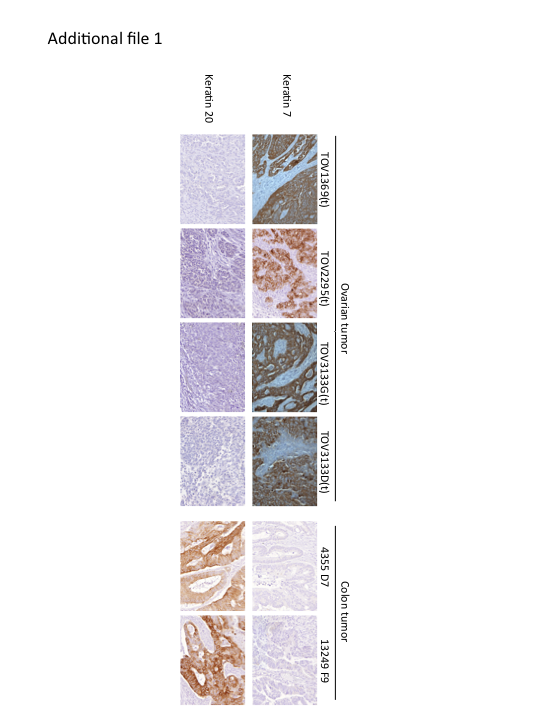

Supplement: Additional file 1 — Keratin 7 and keratin 20 immunohistochemistry in ovarian tumors from which the cell lines were derived. Paraffin embedded colon tumors were used as the negative control for keratin 7, and positive control for keratin 20. Sections were counterstained with hematoxylin and are shown at x40 magnification. [file 1471-2407-12-379-S1.png]

Additional file 2

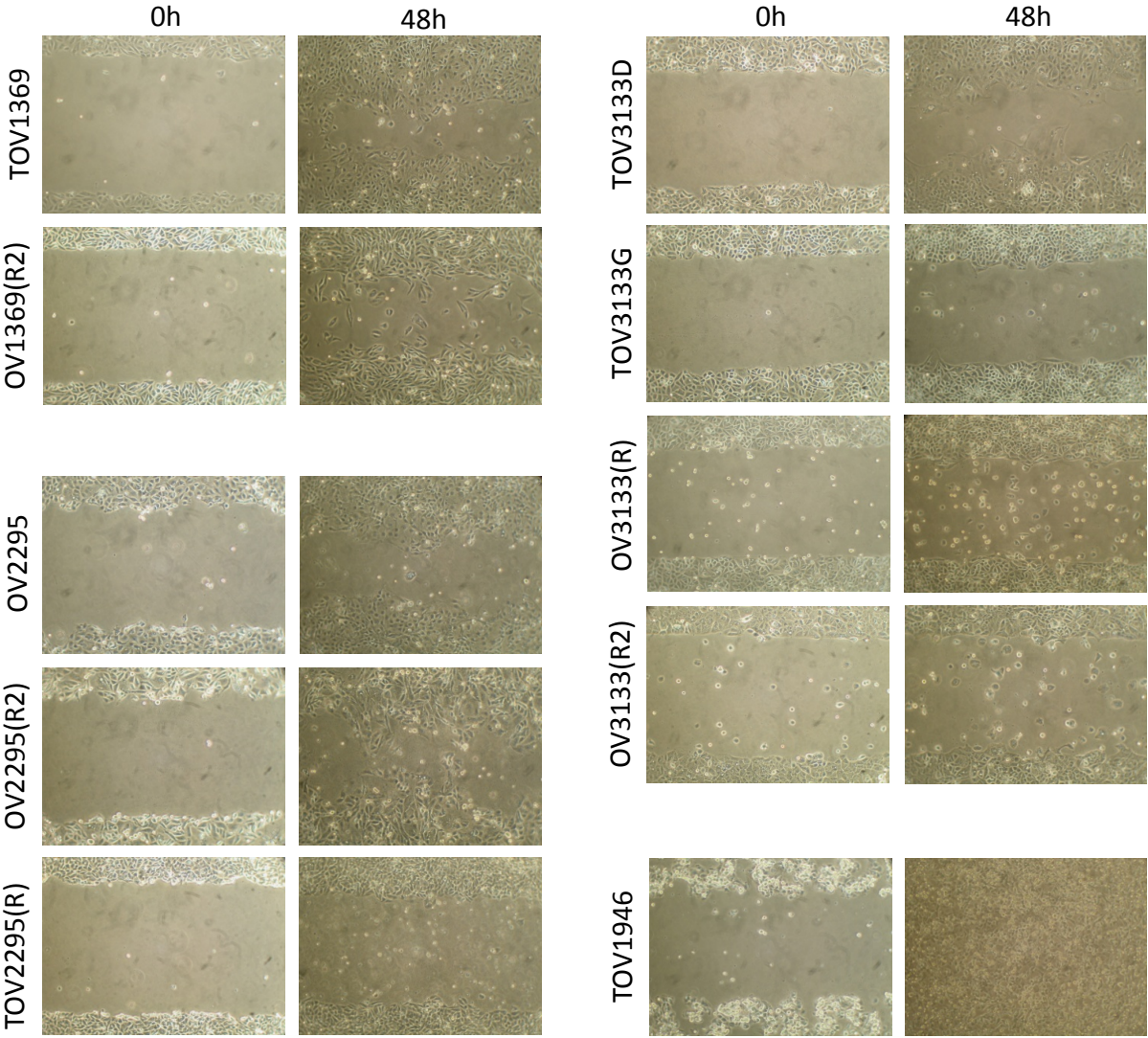

Supplement: Additional file 2 — Images from the migration assay, indicating migration of cells during a 48-hours period. Note the closure of the wound for the TOV1946 cell line after 48 hours. [file 1471-2407-12-379-S2.pdf]

**TOV1369**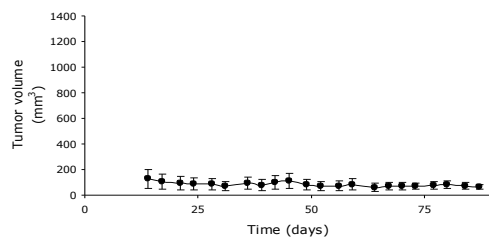**OV1369(R2)**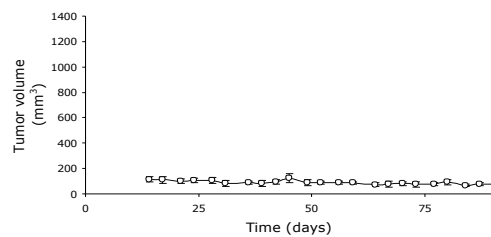**OV2295**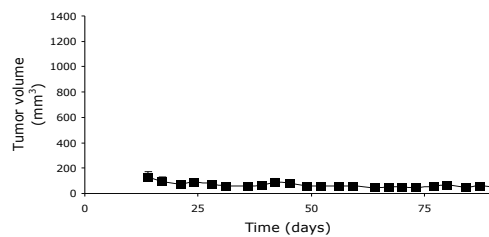**OV2295(R2)**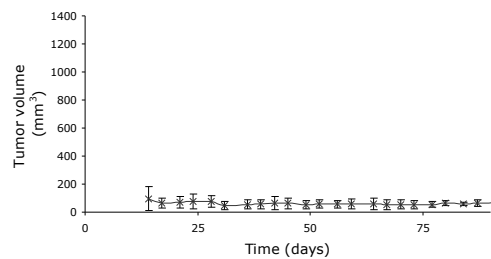**TOV2295(R)**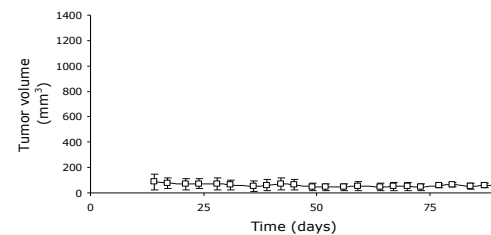**TOV3133G**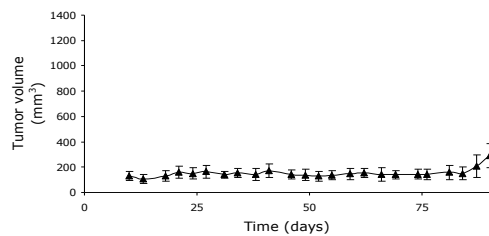**TOV3133D**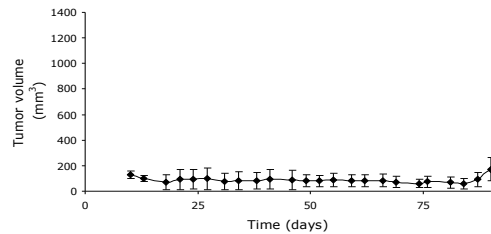**OV3133(R2)**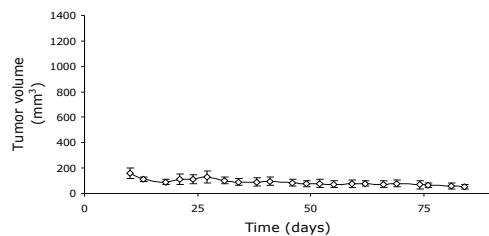**OV3133(R)**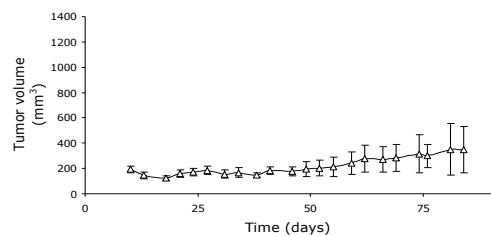**Controls**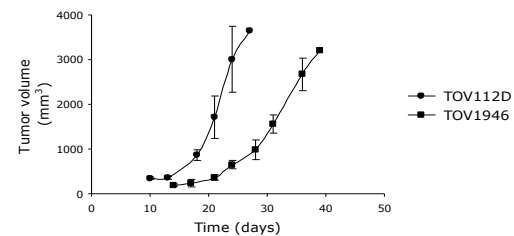

Supplement: Additional file 3 — Tumor growth in SCID mice following injection of specific ovarian cancer cell lines. For each cell line, six SCID mice received subcutaneous injection (5 x 10^6^ cells mixed with Matrigel) (with the exception of five mice for OV1369(R2), and three mice for TOV112D and TOV1946). Graphs represent average ± SD of tumor volume as a function of days following cell injection. Tumor masses were measured at least twice a week (width x length x thickness). Note that the axis for TOV112D and TOV1946 is not of the same scale. Cell lines derived pre-chemotherapy are represented by a closed symbol, cell lines derived post-chemotherapy have an open symbol. [file 1471-2407-12-379-S3.pdf]

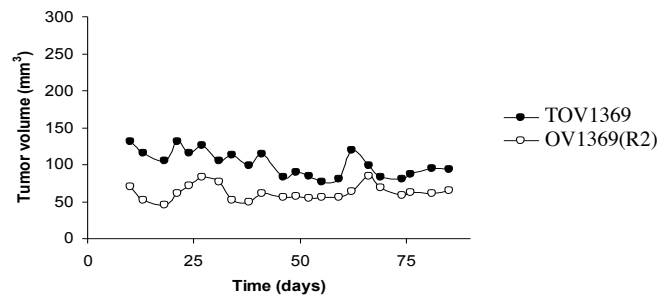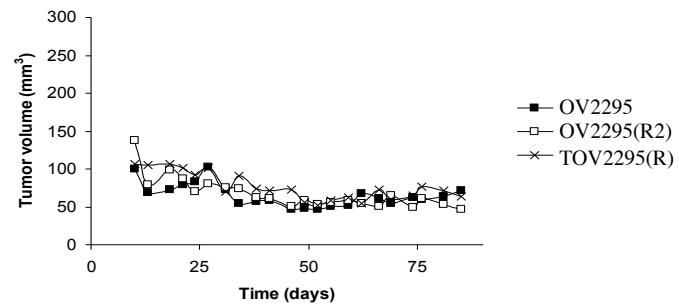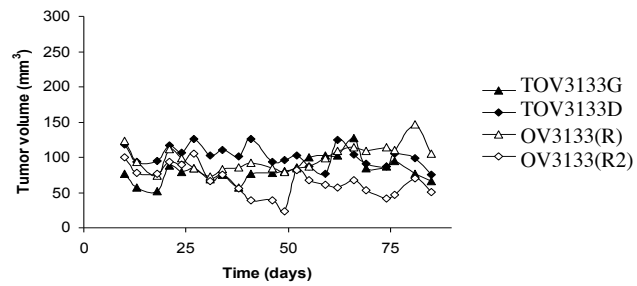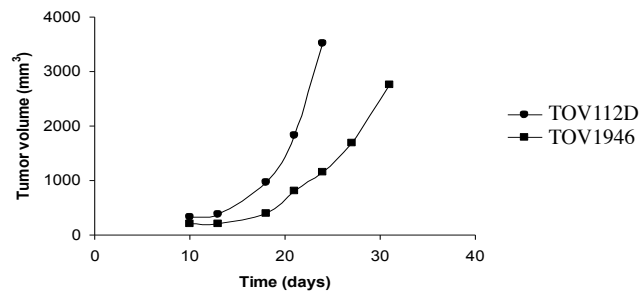

Additional File 4

Supplement: Additional file 4 — Tumor growth in SCID-NOD mice following injection of specific ovarian cancer cell lines. For each cell line, two NOD-SCID mice received subcutaneous injection (5 x 10^6^ cells mixed with Matrigel). Graphs represent average of tumor volume as a function of days following cell injection. Tumor masses were measured at least twice a week (width x length x thickness). Note that the axis for TOV112D and TOV1946 is not of the same scale. Cell lines derived pre-chemotherapy are represented by a closed symbol, cell lines derived post-chemotherapy have an open symbol. [file 1471-2407-12-379-S4.pdf]
